# MR thermometry characterization of a hyperthermia ultrasound array designed using the *k*-space computational method

**DOI:** 10.1186/1475-925X-5-56

**Published:** 2006-10-25

**Authors:** Osama M Al-Bataineh, Christopher M Collins, Eun-Joo Park, Hotaik Lee, Nadine Barrie Smith

**Affiliations:** 1 Department of Bioengineering, The Pennsylvania State University, University Park, PA 16802, USA; 2 Department of Radiology, The Pennsylvania State University, Hershey PA 17033, USA; 3 Graduate Program in Acoustics, The Pennsylvania State University, University Park, PA 16802, USA

## Abstract

**Background:**

Ultrasound induced hyperthermia is a useful adjuvant to radiation therapy in the treatment of prostate cancer. A uniform thermal dose (43°C for 30 minutes) is required within the targeted cancerous volume for effective therapy. This requires specific ultrasound phased array design and appropriate thermometry method. Inhomogeneous, acoustical, three-dimensional (3D) prostate models and economical computational methods provide necessary tools to predict the appropriate shape of hyperthermia phased arrays for better focusing. This research utilizes the *k*-space computational method and a 3D human prostate model to design an intracavitary ultrasound probe for hyperthermia treatment of prostate cancer. Evaluation of the probe includes *ex vivo *and *in vivo *controlled hyperthermia experiments using the noninvasive magnetic resonance imaging (MRI) thermometry.

**Methods:**

A 3D acoustical prostate model was created using photographic data from the Visible Human Project^®^. The *k*-space computational method was used on this coarse grid and inhomogeneous tissue model to simulate the steady state pressure wavefield of the designed phased array using the linear acoustic wave equation. To ensure the uniformity and spread of the pressure in the length of the array, and the focusing capability in the width of the array, the equally-sized elements of the 4 × 20 elements phased array were 1 × 14 mm. A probe was constructed according to the design in simulation using lead zerconate titanate (PZT-8) ceramic and a Delrin^® ^plastic housing. Noninvasive MRI thermometry and a switching feedback controller were used to accomplish *ex vivo *and *in vivo *hyperthermia evaluations of the probe.

**Results:**

Both exposimetry and *k*-space simulation results demonstrated acceptable agreement within 9%. With a desired temperature plateau of 43.0°C, *ex vivo *and *in vivo *controlled hyperthermia experiments showed that the MRI temperature at the steady state was 42.9 ± 0.38°C and 43.1 ± 0.80°C, respectively, for 20 minutes of heating.

**Conclusion:**

Unlike conventional computational methods, the *k*-space method provides a powerful tool to predict pressure wavefield in large scale, 3D, inhomogeneous and coarse grid tissue models. Noninvasive MRI thermometry supports the efficacy of this probe and the feedback controller in an *in vivo *hyperthermia treatment of canine prostate.

## Background

Prostate cancer causes approximately 30,000 deaths among Americans every year with more than 230,000 new patients in 2004 [1]. Most of the patients are elderly and often can not withstand invasive surgical procedures to eradicate the tumor in its early stages [2]. Radiation and hormone therapies are still the treatment of choice for these patients [3]. Thermal treatment has shown to be effective in therapy for different kinds of tumors including prostate cancer [4-8]. Hyperthermia therapy raises the temperature of the tumor and a surrounding margin of normal tissue from the normal body temperature of 37°C to 42–45°C [9-11]. This type of treatment has had success, in either as simultaneous or sequential adjunct to radiation therapy, in enhancing the cytotoxic effect of the radiation therapy [12-15]. Noninvasive ultrasound intracavitary hyperthermia technology is an accepted thermal treatment for prostate cancer [16].

Many previous simulation and design studies of intracavitary ultrasound phased have considered multiple layered media but not necessarily a three-dimensional anatomical prostate model [17-23]. Previous intracavitary ultrasound hyperthermia phased arrays used small cylindrical radiators to conform to the natural contours of large body orifices [24,25]. Simulations of previous hyperthermia and high intensity focused ultrasound (HIFU) phased arrays were accomplished using the Rayleigh-Sommerfeld integral over a set of geometrically superimposed point sources [26]. Homogeneous water-like media were used to simulate pressure field distributions of these arrays [17-20,24,25]. Such simulations, however, do not capture the interaction of ultrasound with inhomogeneous tissue structures. Modeling of ultrasound wave propagation in inhomogeneous three-dimensional (3D) structure or medium over large length scales has become feasible using the *k*-space computational method [27-31]. This method solves the spatial terms of the wave equation by Fourier transformation to the spatial frequency domain, while temporal iterations are performed using a nonstandard finite difference approach using the *k*-t space propagator (where *k *represents the spatial frequency domain and t represents the time domain) [27]. It provides computational improvements over pseudospectral methods, in which the spatial derivatives are evaluated globally by Fourier transformation and wavefields are advanced in time using second order accurate finite differences (leapfrog propagator) [32]. The *k*-space method maintains its accuracy up to a Courant-Friedrichs-Lewy number (CFL = c_0_Δt/Δx, where c_0 _is the sound speed; Δt is the temporal step; Δx is the spatial step) of about 0.4 [27]. However, the pseudospectral method [27] rapidly increases in error for CFL numbers above 0.1. For weak scattering media, the *k*-space method provides similar value for time steps two to three times larger than those required by high order pseudospectral methods [27]. Compared to finite difference computations [33], in which both spatial and temporal second order partial derivatives are solved using second order finite difference computations, the *k*-space method produces practical results for much larger spatial step size. Equivalent accuracy is achieved employing only three points per minimum wavelength using the *k*-space method compared to 14 points per minimum wavelength for the finite difference equation using the same criterion. For 3D calculations, this reduction in the spatial size reduces the storage requirements for the *k*-space computations compared to finite difference method by 98% [27].

Noninvasive magnetic resonance imaging (MRI) thermometry is helpful in monitoring and controlling hyperthermia treatment of the prostate gland [21,34-36]. It is important in this therapy to keep the temperature of the healthy tissue below the targeted temperature of the cancerous volume. A feedback control system is useful in maintaining the targeted tissue within the required thermal dose for cytotoxicity (43°C for 30 minutes) [37]. This research focuses on acoustical modeling of a 4 × 20 element hyperthermia phased array, exposimetry testing, and *ex vivo *and *in vivo *evaluation of the probe utilizing MRI thermometry.

## Methods

### Phased array design and the k-space acoustic modelling

**Figure 1:**
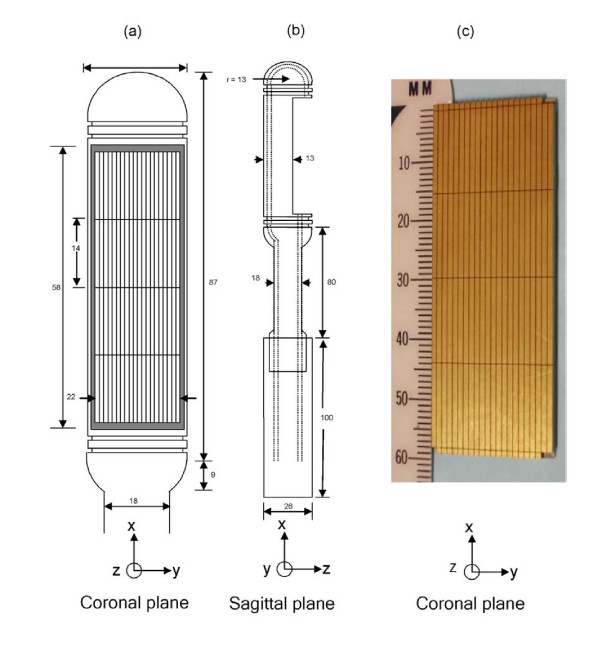
**Hyperthermia phased array**. Two diagrams and a photograph showing the transrectal intracavitary ultrasound probe in (a) coronal view (xy-plane), (b) sagittal view (xz-plane), and (c) an actual photograph of the diced hyperthermia array.

Figure 1 shows several views (xy-plane is the coronal plane; yz-plane is the transverse or axial plane; and xz-plane is the sagittal plane) of the 4 × 20 hyperthermia phased array in its intracavitary housing. The array consists of four segments of planar phased arrays; each segment consists of 20 elements with a 1 × 14 mm sub-element dimension. This hyperthermia phased array enables focusing of the pressure beam in the propagation (z-direction) and the azimuthal (y-direction) directions and enables spreading of the focal region in the volume in front of the array (x-direction). Electronic phasing of the elements that make up each segment allows for steering of the beam in the azimuth direction and adjustment of the depth of focus in the propagation direction. The focusing mechanisms permit varying the heating in the prostate gland to achieve uniform thermal dose to the targeted volume. Simulation of the exact pressure wavefield in the prostate gland requires building anatomically and acoustically accurate inhomogeneous human prostate model.

**Figure 2:**
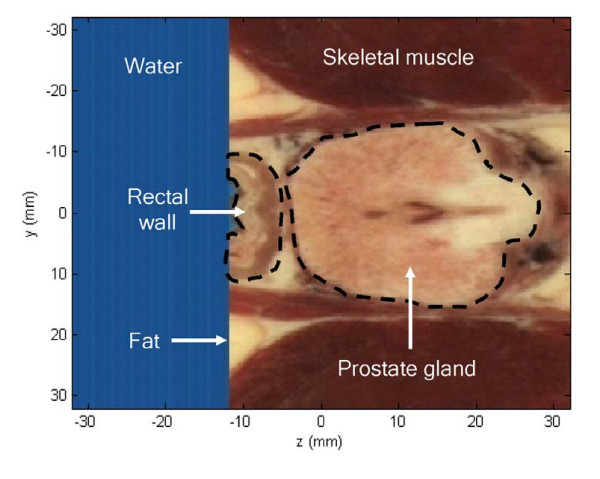
**The prostate model**. From the Visible Human Project^®^, a photographic image of a prostate slice shows a transverse (axial, yz-plane) cross section of the prostate gland.

An anatomically and acoustically accurate 3D prostate model was created using photographic images from the Visible Human Project^® ^library (U.S. National Library of Medicine, Bethesda, MD). Figure 2 shows a transverse (yz-plane) photographic slice of this model. It has 64 × 64 mm dimensions with a 0.25 mm grid size. The prostate gland, the rectal wall, the skeletal muscle, the fat tissue, and the added water-like medium in the rectum are marked and labeled in the figure. The optical parameters that define the fractional fat, connective, glandular and muscle soft tissues of each pixel were used to build a three-dimensional acoustical model. The 3D photographic data permitted development of three sets of 3D acoustical data: sound speed, density and absorption parameters[27,38]. More details of the mapping procedure are presented elsewhere [39]. The prostate model was used to simulate the pressure distribution of the hyperthermia phased array by means of the *k*-space computational method.

The *k*-space method was used to study pressure beam formation of the designed phased array through the prostate model. The linear acoustic wave equation was used for the simulation:

∇⋅(1ρ(x,y,z)∇p(x,y,z,t))−1ρ(x,y,z)c2(x,y,z)∂2p(x,y,z,t)∂t2=α(x,y,z)ρ(x,y,z)c2(x,y,z)∂p(x,y,z)∂t
 MathType@MTEF@5@5@+=feaafiart1ev1aaatCvAUfKttLearuWrP9MDH5MBPbIqV92AaeXatLxBI9gBaebbnrfifHhDYfgasaacH8akY=wiFfYdH8Gipec8Eeeu0xXdbba9frFj0=OqFfea0dXdd9vqai=hGuQ8kuc9pgc9s8qqaq=dirpe0xb9q8qiLsFr0=vr0=vr0dc8meaabaqaciaacaGaaeqabaqabeGadaaakeaacqGHhis0cqGHflY1cqGGOaakdaWcaaqaaiabigdaXaqaaGGaciab=f8aYjabcIcaOiabdIha4jabcYcaSiabdMha5jabcYcaSiabdQha6jabcMcaPaaacqGHhis0cqWGWbaCcqGGOaakcqWG4baEcqGGSaalcqWG5bqEcqGGSaalcqWG6bGEcqGGSaalcqWG0baDcqGGPaqkcqGGPaqkcqGHsisldaWcaaqaaiabigdaXaqaaiab=f8aYjabcIcaOiabdIha4jabcYcaSiabdMha5jabcYcaSiabdQha6jabcMcaPiabdogaJnaaCaaaleqabaGaeGOmaidaaOGaeiikaGIaemiEaGNaeiilaWIaemyEaKNaeiilaWIaemOEaONaeiykaKcaamaalaaabaGaeyOaIy7aaWbaaSqabeaacqaIYaGmaaGccqWGWbaCcqGGOaakcqWG4baEcqGGSaalcqWG5bqEcqGGSaalcqWG6bGEcqGGSaalcqWG0baDcqGGPaqkaeaacqGHciITcqWG0baDdaahaaWcbeqaaiabikdaYaaaaaGccqGH9aqpdaWcaaqaaiab=f7aHjabcIcaOiabdIha4jabcYcaSiabdMha5jabcYcaSiabdQha6jabcMcaPaqaaiab=f8aYjabcIcaOiabdIha4jabcYcaSiabdMha5jabcYcaSiabdQha6jabcMcaPiabdogaJnaaCaaaleqabaGaeGOmaidaaOGaeiikaGIaemiEaGNaeiilaWIaemyEaKNaeiilaWIaemOEaONaeiykaKcaamaalaaabaGaeyOaIyRaemiCaaNaeiikaGIaemiEaGNaeiilaWIaemyEaKNaeiilaWIaemOEaONaeiykaKcabaGaeyOaIyRaemiDaqhaaaaa@9E42@

where, ∇·( ) is the spatial divergence operator; ∇( ) is the spatial gradient operator; *ρ*(*x*, *y*, *z*) is the spatially dependent density (kg/m^3^); *c*(*x*, *y*, *z*) is the spatially dependent sound speed (m/s); *p*(*x*, *y*, *z*, *t*) is the spatially and temporally dependent pressure (Pa); *α*(*x*, *y*, *z*) is the spatially dependent absorption coefficient (s^-1^, the absorption in dB/m equals to 20 × log_10_(e) × *α*(*x*, *y*, *z*)/(2c_0_) [39]). All absorption effects (viscous, heat conduction and internal molecular processes losses) were represented by a single absorption coefficient which was equivalent to the inverse of a spatially dependent relaxation time [40]. The *k*-t propagator was used to solve for the propagation in the inhomogeneous prostate model after setting both initial and boundary conditions [27]. The dimensions of the model were 64 × 64 × 46 mm with 0.25 mm spatial step size. It was composed of 257 × 257 × 185 discrete points. The temporal step size was 0.082 μs. A tapered absorption boundary layer, all around the model, was created to prevent wave wrapping from side to side and to prevent reflection of the waves at the boundaries. This layer is mathematically described elsewhere [39]. A single segment of the phased array was incorporated in the acoustical model for simulation purposes. Virtual elements with 1 × 14 mm dimensions were integrated in the simulation. The established grid size of 0.25 mm for the model limited the effective kerf width (dice thickness) of the array to this number. Each sub-element added to the overall virtual source that induced pressure to the surrounding media, depending on the acoustical parameters of each point of the model. All points that related to a specific element were driven temporally in a sinusoidal fashion with a 1.2 MHz resonance frequency and a particular phase shift that compensated for its path length to a specific target. Greater details regarding the simulations with respect to the design of the array are described elsewhere [29-31].

**Figure 3:**
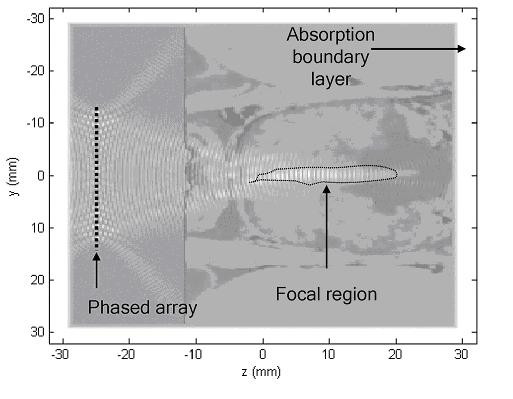
**The *k*-space simulation**. A gray scaled image showing the simulated normalized pressure squared distribution of a single segment of the hyperthermia phased array using the k-space computational method. The results are shown for the central transverse plane of the 3D prostate model. The image shows a two-layered gray scale photograph for the central plane showing a background layer of the absorption distribution and the normalized squared pressure distribution on top of it while focusing axially 40 mm away from the face of the array.

Figure 3 shows an axial two-layered gray-scale image of both the *k*-space simulated normalized pressure squared distribution and the absorption variations of the previously shown slice in Figure 2. The dimensions of the slice are 64 × 64 mm. The white colored boundaries of the image represent the tapered absorption layer, which prevents the reflection and wrapping of the ultrasound waves at the boundaries. The phased array is located 5 mm away from the absorbing boundary layer. It is coupled to the rectal wall through the water medium. The pressure squared distribution is represented by the white colored waves on top of the absorption gray-scale distribution. Inhomogeneous tissue composition through the rectal wall and the prostate gland causes irregularity of the focused beam. The acoustic energy is focused inside the prostate gland 40 mm away from the phased array.

### Hyperthermia phased array exposimetry testing

The hyperthermia phased array system was tested using an in-house automated exposimetry system based on the American Institute of Ultrasound in Medicine and National Electrical Manufacturers Association (AIUM/NEMA) guidelines [41]. The array was submerged in an anechoic tank (122 × 51 × 53 cm) filled with degassed distilled water. A needle-type hydrophone (precision Acoustics Ltd., Dorchest, UK) was placed perpendicular to the face of the transducer to measure pressure field values at discrete points. While focusing the acoustical energy 40 mm axially away from the face of the transducer, seven scans were acquired in the propagation direction for a single segment of the phased array. The average values of these scans were compared to *k*-space and Rayleigh-Sommerfeld simulation results.

Unlike *k*-space computations, the Rayleigh-Sommerfeld simulations computed the pressure distribution produced by a single segment of the phased array by summing the pressure contributions of individual simple sources along the extracted lines. The kerf width was 0.12 mm and the simulations were performed in water medium without the inclusion of the absorption term.

**Figure 4:**
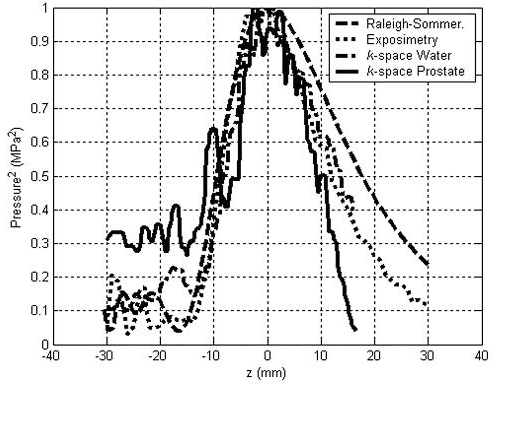
**Exposimetry results**. Normalized pressure squared distribution in linear scans through the focal point in the propagation direction showing the averaged exposimetry results compared to conventional Rayleigh-Sommerfeld and *k*-space simulation results.

Figure 4 shows the normalized pressure squared of a line that crosses the focal point in the z-direction. The mean exposimetry results are compared to the Rayleigh-Sommerfeld, *k*-space in water medium, and *k*-space in prostate model simulations. The *k*-space simulation in the prostate model shows that the inhomogeneous tissue structure of the rectal wall and the prostate gland cause focusing aberration and elevation of the pressure values (< -3 dB) within the nearfield region compared to exposimetry and other simulations. Rapid decrease in the pressure values of the *k*-space prostate simulation is due to the relatively high absorption values of this extracted axial line which mostly composed of connective tissue with absorption values of 110 dB/m. Both exposimetry results and *k*-space water simulation results show acceptable agreement within a 9% calculated error when comparing the -3 dB widths of the focal volume. Rayleigh-Sommerfeld simulation shows deviation of the results compared to the *k*-space simulations and exposimetry results. This deviation is due to performing the calculations of the pressure values without the inclusion of absorption effects.

### MRI thermometry methods

**Figure 5:**
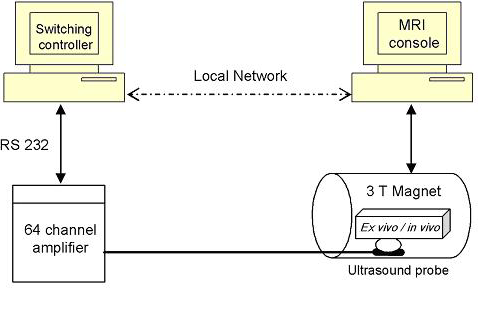
**Hyperthermia setup**. A sketch shows the setup of hyperthermia experiments using MRI thermometry.

*Ex vivo *and *in vivo *hyperthermia evaluations of the probe were made using MRI thermometry and a switching feedback controller. Figure 5 shows the setup for the hyperthermia experiments. A personal computer used as a switching temperature controller was connected via an RS232 serial port to the digital power amplifier (UDS 2050PA, Advanced Surgical Systems, Inc. Tucson, AZ) and to the console of the magnetic resonance imaging system (3 Tesla MEDSPEC S300, Bruker BioSpin, Ettlingen, Germany). The ultrasound transrectal probe was coupled to either *ex vivo *bovine tissue samples (phantom) or *in vivo *canine prostate gland using an inflated bolus of circulated water. The transducer was connected to the driving power amplifier. Water hoses were connected to a water pump (Cole-Parmer Instrument Company, Barrington, IL) via a bubble trap chamber and air hoses were connected to an air pump. Depending on feedback temperature values, the switching controller adjusted the driving power of each ultrasound channel by signaling the power amplifier system on and off. Temperature values were calculated from the phase shift of the acquired MRI images as follows [42]:

ΔT = Δφ/(α γ TE B_0_)

where, ΔT is the relative temperature (°C); Δφ is the phase difference (rad); α is the temperature dependent chemical shift (-0.00909 ppm/°C); γ is the gyromagnetic ratio (rad/s.T); TE is the echo time (s), and B_0 _is the magnetic field (T). A spoiled gradient (SPGR) echo sequence was used to acquire thermal images for the feedback controller. More details are presented elsewhere [36].

For *ex vivo *experiments, the transrectal probe and its bolus were held close to the bovine tissue sample and the whole apparatus was inserted in the RF head coil and was placed in the uniform static magnetic field and gradient coils. A base line image was produced using these parameters: repetition time TR = 100 ms, echo time TE = 15 ms, flip angle = 30°, data matrix = 64 × 64, field of view (FOV) = 12 × 12 cm, and slice thickness = 4 mm. The ultrasound transducer was excited for 5 minutes before acquiring another image. Phase difference values, between base image (before driving the transducer) and an image after five minutes of driving the transducer, were used to calculate temperature variations in the selected slice. The MRI-derived average temperature of a 2 × 3 pixel region was used as an input to the controller. *In vivo *animal experiments were conducted with procedures approved by the Penn State Institutional Animal Care and Use Committee (IACUC). A mongrel-type canine (3 years old, 10 kg) was anesthetized with Telazol (100 mg/ml, reconstituted with Tiletamine hydrochloric acid and Zolazepam hydrochloric acid, Fort Dodge Animal Health, Fort Dodge, IA) and was placed inside the magnet. The rectum of the dog was manually cleaned and was filled with ultrasound gel using a syringe. The transrectal probe was inserted in the rectum facing the prostate gland. The vital readings of the animal were periodically checked and recorded. MRI images were acquired to help aligning both the prostate gland and the phased array perpendicularly to each other. A baseline image was produced before driving the phased array. Another image was produced five minutes after driving the transducer. These images were used to calculate thermal distribution through the prostate gland. A smaller region of interest (ROI) area inside the prostate was used to average the temperature value and to feedback the controller system. Each controlled hyperthermia experiment was executed for 20 minutes.

## Results

### *Ex vivo *results

**Figure 6:**
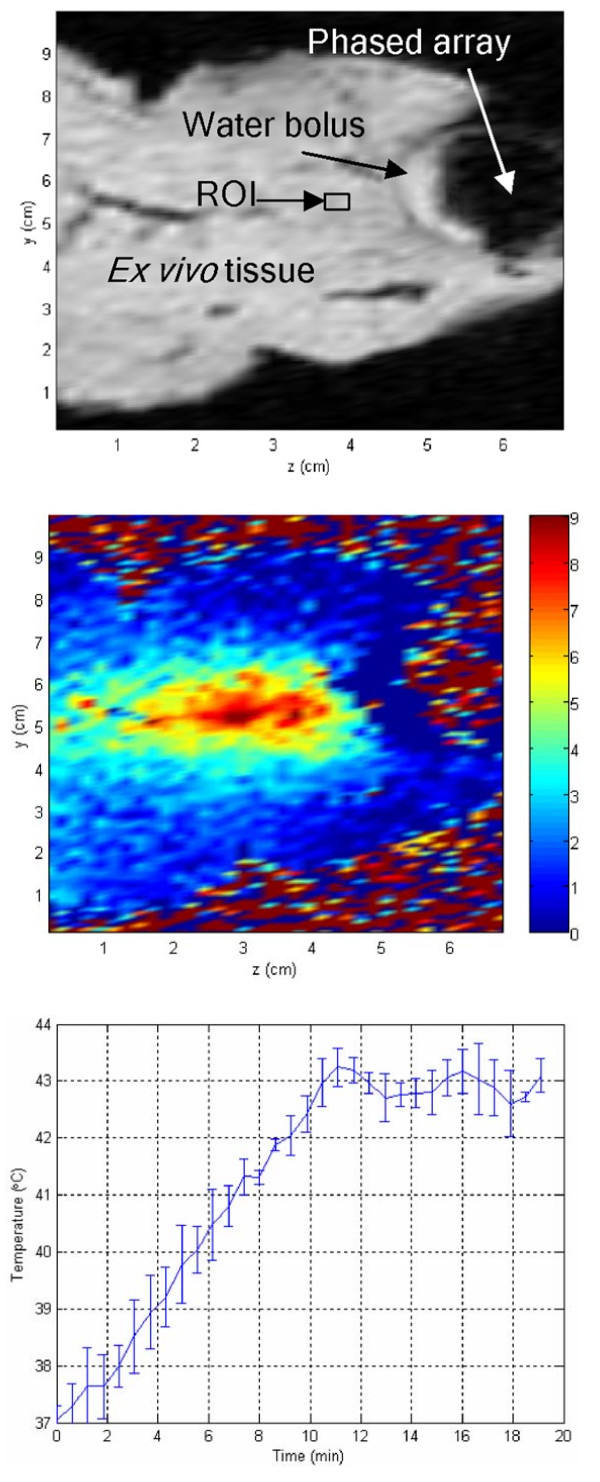
***Ex vivo *MRI hyperthermia**. (a) An MRI image of a selected bovine slice showing the focused transducer cross sectional view. (b) The thermal image after driving the transducer for five minutes. The color bar relates relative temperature values in °C. (c) *Ex vivo *controlled hyperthermia results using MRI thermometry and the switching controller.

Hyperthermia controlled *ex vivo *experiments using MRI thermometry were conducted for 20 minutes. Figure 6a shows a transverse MRI image of the coupled transrectal probe to an *ex vivo *bovine tissue sample with dimensions of 100 × 70 × 4 mm. Water bolus provides good coupling medium between the active elements of the array and the tissue. Figure 6b shows the calculated relative thermal distribution after driving the transducer for five minutes. The color bar illustrates the relative temperatures in °C. Ultrasound energy is concentrated 20 mm away from the face of the transducer and is spread axially for 30 mm. Temperature increases vary from 5°C to 9°C within the focal region. The water bolus temperature is kept constant during heating period. Figure 6c shows the results of *ex vivo *controlled hyperthermia using MRI thermometry. Aiming at 6°C relative rise, the averaged temperature of the region of interest is raised 5.9 ± 0.38°C in 9.5 ± 0.26 minutes and is kept till the end of the experiment. The solid continuous line represents averaged temperature values of seven different experiments. The standard error bars are shown at discrete points of 30 seconds intervals.

### *In vivo *results

**Figure 7:**
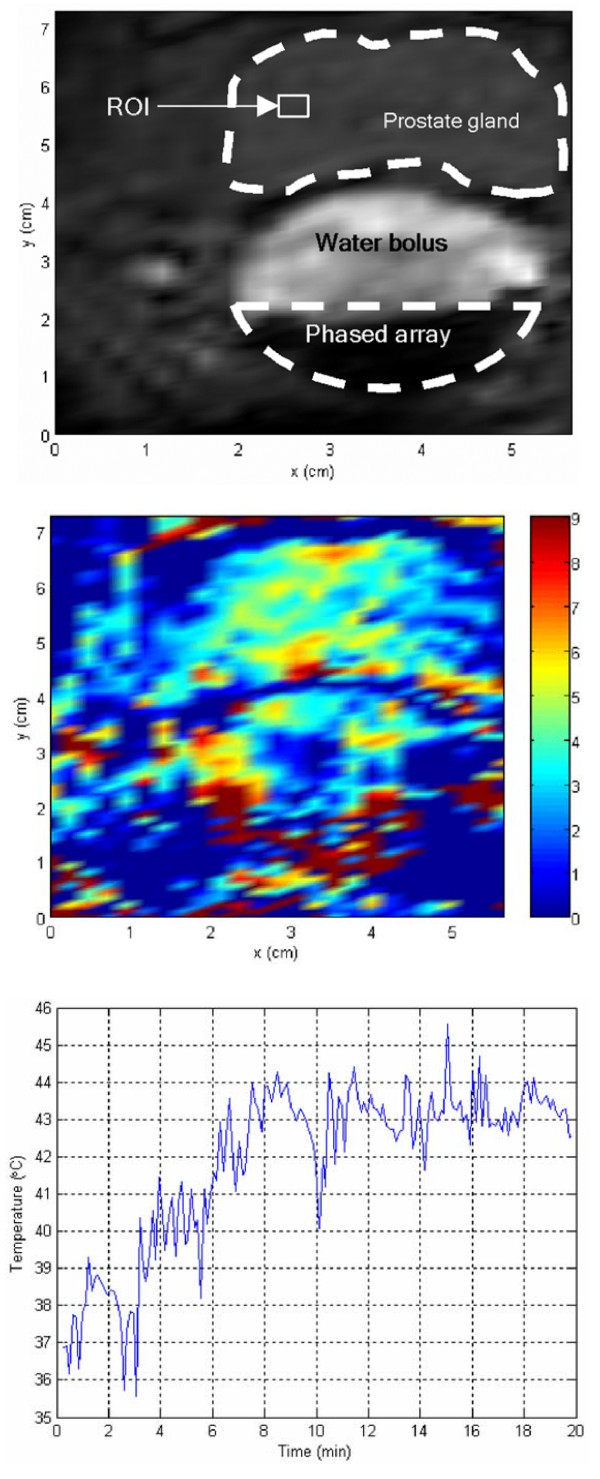
***In vivo *MRI hyperthermia**. *In vivo *canine prostate hyperthermia results. (a) MRI image of a canine prostate gland showing the focused transducer and the water bolus. (b) Relative thermal distribution image produced after driving the transducer for five minutes. The color bar shows the temperature values in °C. (c) Controlled hyperthermia results for 20 minutes.

Figure 7a shows a transverse MRI image of the transrectal probe coupled via the pressurized water bolus to a canine prostate gland. The dimensions of this slice are 70 × 60 × 4 mm. Water bolus provides good coupling medium between the array and the prostate. Figure 7b shows the calculated relative thermal image after driving the transducer for five minutes. The color bar illustrates the relative temperatures in °C. Ultrasound energy is spread through the prostate region. Relative temperature values vary from 3°C to 6°C within the prostate gland. Circulated water temperature within the bolus is intended to be homogeneous and close to zero. However, inhomogeneous distribution of temperature throughout the bolus is due to slower flow of the pumped water. Averaged temperature of a small ROI area of 2 × 3 pixels within the prostate gland is used as a feedback value for the controller. Figure 7c shows the results of *in vivo *controlled hyperthermia. With a desired relative temperature of 6°C, results show that the temperature of the ROI is risen 6.1 ± 0.80°C in 6.3 minutes and is maintained approximately steady till the end of the experiment. The solid line represents discrete temperature values every 7 seconds.

## Discussion

The 4 × 20 element phased array provides focusing of the pressure wavefield within the prostate gland. The spreading of the focal volume in the length of the array (the elevation-direction or x-direction) is achieved by recruiting more segments to heat the whole prostate gland. Rayleigh-Sommerfeld and *k*-space simulations help in predicting the appropriate dimensions of the array. Good agreement between exposimetry results and the simulated *k*-space results was achieved. As an example, the -3 dB distance of the focal volume in the propagation direction (z-direction) is off by 9% between exposimetry and *k*-space simulations. Hyperthermia experiments of the focused probe were compared to a 16-element unfocused transducer [34]. With a desired relative temperature of 6°C, the controlled hyperthermia experiments show that the steady temperature of the ROI is maintained at 6.5 ± 0.93°C and 42.8 ± 1.44°C for *ex vivo *and *in vivo *experiments, respectively. Compared to unfocused transducers, however, the focused transducer has the ability of focusing acoustic energy in targeted tissue and at the same time has the ability to steer the beam for better treatment. Unfocused transducer spreads the energy in a fan-shaped profile in the tissue in front of the transducer. *In vivo *canine prostate hyperthermia trial proves the usefulness of the focused probe in prostate treatment. Blood flow can be considered a natural cooling system that works against temperature elevation within the prostate. The tested probes are capable of counteracting the effect of blood cooling while keeping the targeted volume within the required biological thermal dose.

Tissue-ultrasound interaction requires simulation of the ultrasound perturbations produced from phased arrays instead of summing the pressure contribution of geometrically superimposed simple sources. This requirement becomes feasible using the *k*-space computational method which provides economical and accurate simulation tool for large scale, coarse grid and inhomogeneous tissue models. Simulation results of the *k*-space are in good agreement with actual exposimetry results.

The 4 × 20 phased array intentionally spreads the focal volume in the length of the array (x-direction) and allows for varying in the width of the array (y-direction) while changing the depth of the focusing in the axial direction (z-direction). These variable parameters allow better thermal targeting of the whole prostate gland and the seminal vesicles. Controlling the temperature of a single point within the targeted volume helps in delivering the required clinical thermal dose into the targeted volume while maintaining surrounded desired tissue. Noninvasive MRI thermometry is essential in monitoring and controlling of thermal treatment of the prostate cancer. Ultimately, this research has benefited from two non-invasive technologies to help develop treatment for prostate cancer in conjunction with classical therapeutic modalities.
